# A CNN-Based Deep Learning Approach for SSVEP Detection Targeting Binaural Ear-EEG

**DOI:** 10.3389/fncom.2022.868642

**Published:** 2022-05-19

**Authors:** Pasin Israsena, Setha Pan-Ngum

**Affiliations:** ^1^National Electronics and Computer Technology Center (NECTEC), National Science and Technology Development Agency (NSTDA), Pathumthani, Thailand; ^2^Department of Computer Engineering, Faculty of Engineering, Chulalongkorn University, Bangkok, Thailand

**Keywords:** brain-computer interface, SSVEP, CNN, ear-EEG, binaural

## Abstract

This paper discusses a machine learning approach for detecting SSVEP at both ears with minimal channels. SSVEP is a robust EEG signal suitable for many BCI applications. It is strong at the visual cortex around the occipital area, but the SNR gets worse when detected from other areas of the head. To make use of SSVEP measured around the ears following the ear-EEG concept, especially for practical binaural implementation, we propose a CNN structure coupled with regressed softmax outputs to improve accuracy. Evaluating on a public dataset, we studied classification performance for both subject-dependent and subject-independent trainings. It was found that with the proposed structure using a group training approach, a 69.21% accuracy was achievable. An ITR of 6.42 bit/min given 63.49 % accuracy was recorded while only monitoring data from T7 and T8. This represents a 12.47% improvement from a single ear implementation and illustrates potential of the approach to enhance performance for practical implementation of wearable EEG.

## Introduction

Brain–computer interfaces (BCIs) provide direct communication between the brain and external devices without relying on peripheral nerves and muscle tissue (Wolpaw et al., 2000). This can be useful in a number of scenarios, for example, in cases when users have ALS or locked-in syndrome. To enable this, brain imaging techniques are used to analyze brain activities before translating them into device's commands. Among existing techniques are functional magnetic resonance imaging (fMRI) (Suk et al., 2016), functional near-infrared spectroscopy (fNIRS) (Naseer and Hong, 2015), magnetoencephalography (MEG) (Mellinger et al., 2007), and electroencephalography (EEG). Due to its relatively low cost, portability, and high temporal resolution, EEG is one of the most widely used non-invasive methods in BCI. To generate the output commands of the EEG-based BCI, several types of physiological paradigms have been considered such as motor imagery (MI) (Wolpaw et al., 1991), P300 (Farwell and Donchin, 1988), steady-state visual evoked potential (SSVEP) (Cheng et al., 2002), and steady-state auditory evoked potential SSAEP (Van Dun et al., 2007; Kim et al., 2012). SSVEP, in particular, has gained a lot of attention for its characteristics of less training, high classification accuracy, and high information transfer rate (ITR) (Wolpaw et al., 2002).

SSVEPs are periodic responses elicited by the repetitive fast presentation of visual stimuli. They are mainly generated in the occipital area, operate typically at frequencies between ~1 and 100 Hz, and can be distinguished by their characteristic composition of harmonic frequencies (Herrmann, 2001). Different target identification methods have been considered for detecting SSVEPs in BCIs (Wang et al., 2008; Vialatte et al., 2009; Gao et al., 2014). Originally, power spectrum density analysis (PSDA)-based methods such as fast Fourier transform (FFT) were widely used for frequency detection with single-channel EEGs (Cheng et al., 2002; Wang et al., 2006). More recently, spatial filtering methods including canonical correlation analysis (CCA) (Lin et al., 2007) and common spatial pattern (CSP) (Parini et al., 2009) have been applied to achieve more efficient target identification results. The CCA-based method was first developed for the frequency detection of SSVEPs in 2007 (Lin et al., 2007). It performs canonical correlation analysis between multi-channel EEG signals and predefined sinusoidal reference signals at stimulation frequencies and identifies the target frequency based on the canonical correlation values. The CCA method, in particular, has been widely used (Bin et al., 2009; Wang et al., 2010, 2011; Chen et al., 2014a) because of its high efficiency, ease of implementation, and the fact that it does not require calibration. To improve the performance, further studies of VEP-based BCIs have suggested for incorporating individual calibration data in CCA-based detection to reduce misclassification caused by the spontaneous EEG signals (Bin et al., 2011; Zhang et al., 2011, 2013, 2014; Chen et al., 2014b; Nakanishi et al., 2014; Wang et al., 2014), as the phase and amplitude of the fundamental and harmonic components from each subject are different. The most widely used methods for these enhanced CCA include the following: combination method-CCA (Nakanishi et al., 2015), individual template CCA (IT-CCA) (Wang et al., 2014), and more recently proposed task-related components analysis (TRCA) (Nakanishi et al., 2018). Most recent work on SSVEP includes advanced techniques such as filter bank-driven multivariate synchronization algorithm (Qin et al., 2021) and multivariate variational mode decomposition-informed canonical correlation analysis (Chang et al., 2022).

Machine learning is a branch of artificial intelligence (AI). Without being explicitly programmed, the focuses are on the use of data and algorithms for the computer to imitate the way that humans learn and gradually improve its accuracy. Machine learning has been used to solve many real-world problems, the emerging of deep learning which is a class of machine learning algorithms that uses multiple layers to progressively extract higher-level features from the raw input, in particular, has recently led to an explosive grown in the field. Convolutional neural networks, or CNNs, are artificial neural networks that can learn local patterns in data by using convolutions as their key component. CNNs vary in the number of convolutional layers, ranging from shallow architectures with just one convolutional layer such as in a successful speech recognition (Abdel-Hamid et al., 2014) to deep CNN (DCNN) with multiple consecutive convolutional layers (Krizhevsky et al., 2012). As CNNs do not strictly require feature extraction before processing compared to other machine learning techniques such as linear discriminant analysis (LDA), support vector machine (SVM), or k-nearest neighbor (KNN), they can combine automatic feature extraction and classification to form an end-to-end decoding method which is very attractive for practical considerations. CNNs have indeed been successfully applied in fields such as computer vision and speech recognition medical image analysis. In general, a good review on how deep learning has been studied and applied in non-invasive brain signals, and its potential applications can be found at Zhang et al. (2021).

In terms of CNN and SSVEP, Podmore et al. proposed a deep convolutional neural networks (DCNNs) architecture to classify an open-source SSVEP dataset which included 40 stimuli for speller task with 87% offline accuracy using the period of data observation (window length) of 6 s (Podmore et al., 2019). In Kwak et al. (2017), a 2-D map (channels x frequencies) of SSVEP data was used as the input to classify up to five SSVEP frequencies using a multi-channel EEG headset. To control an exoskeleton in an ambulatory environment, they achieved an accuracy above 94%, using a data length of 2s, and surpassed CCA performances. In Nguyen et al. (2016), a one-dimensional DCNN was applied to create a virtual keyboard using a single-channel SSVEP-based BCI. An accuracy above 97% was achieved with a 2s data length and close to 70% with a 0.5s window. Their CNN results also surpassed CCA.

One of the major challenges for BCI to be widely adopted has been to improve its practicality. Scalp-based EEG, especially the conventional wet-electrode versions, requires the use of conductive gel to enable a connection between the electrodes and the scalp. As the recording quality degrades considerably once the gel dries out, this makes them unsuitable for 24-h use. On top of the preparation time necessary before for each wearing, the use of electrode gel also leaves residue for which users need to wash their hair at the end of each recording session, adding further inconveniences. Dry electrodes remove some of the inconveniences but at the expense of new issues such as increased susceptibility to artifacts (Kam et al., 2019; Marini et al., 2019). A number of research teams have since turned their attention to the concept of ear-EEG. Ear-EEGs are EEG devices that acquire signals around ears or in the external ear canal. Not only these devices can potentially bring benefits in terms of convenience, unobtrusiveness, and mobility, but as people are already accustomed to hearables devices such as the wireless headphones or hearing aids in everyday life, this could potentially lead to a much wider acceptance.

The first ear-EEG, which was an in-ear device with two-channel electrode, was introduced by Looney (Looney et al., 2011). Improvements have since been reported (Looney et al., 2012; Kidmose et al., 2013; Kappel et al., 2014; Mikkelsen et al., 2015). In general, a good match was observed between the ear-EEG and on-scalp responses even though the ear-EEG had lower absolute amplitudes than on-scalp EEG. It was also shown that the degree of correlation between the on-scalp and ear-EEG electrodes was higher, especially for on-scalp electrodes placed near the temporal region (T7, T8) (Looney et al., 2012). Alternatively, another major approach in ear-EEG has been the use of multi-channel EEG placed around the ear, for which many researches (Bleichner et al., 2016; Mirkovic et al., 2016; Bleichner and Debener, 2017) have been based on the cEEGrid devices proposed in Debener et al. (2015). Potentially, there are a number of applications, clinical and nonclinical, for which a small number of electrodes are sufficient, and for which a fully wearable recording platform is a prerequisite, including a hearing aid (Mirkovic et al., 2016; Christensen et al., 2018), sleep monitoring (Nguyen et al., 2016; Goverdovsky et al., 2017), biometric identification (Nakamura et al., 2018), epilepsy detection system (Gu et al., 2017), and fatigue estimation (Looney et al., 2014a).

Combining an SSVEP paradigm with the ear-EEG has been the theme explored in recent researches (Looney et al., 2012, 2014a; Kidmose et al., 2013; Lee et al., 2014; Goverdovsky et al., 2016; Kappel and Kidmose, 2017; Kappel et al., 2019). Wang et al. were the first to conduct offline and online experiments to evaluate the feasibility of decoding SSVEP from the occipital brain region compared to non-hair-bearing areas including the face, behind ears, and neck areas (Wang et al., 2012; Wang Y. T. et al., 2017). The results showed best SNRs of SSVEP were obtained from the occipital areas as expected, with behind-the-ear better than neck and face areas illustrating the potential use of ear-EEG for SSVEP-based BCI. The ear-EEG has indeed been proven to be capable of collecting evoked brain activities such as SSVEP. However, a long distance between the visual cortex and the ear makes the signal-to-noise ratio (SNR) of SSVEPs acquired by earpieces relatively low. For example, Kidmose et al. (2013) proposed an earplug-type ear-EEG electrode. With three classes (10, 15, and 20 Hz) of SSVEP, SNR was measured in comparison with scalp-EEG. On average, SSVEP qualities of ear-EEG at the first harmonic frequencies were found to decrease from 30 to 10 dB. Looney et al. (2014b) also found the SSVEP performance to decrease by ~50% (i.e., capacity ratios for scalp- and ear-EEG based on the estimated SNR and independent of the stimulus presentation) using ear-EEG with two LED visual stimuli (i.e., 15 and 20 Hz). The level of performance reduction was agreeable with Wang's report (Wang et al., 2012).

It is interesting to note that all ear-EEG studies except multi-channel cEEGrid-based ones have looked at single ear measurement. As people are accustomed to two-ear wearing such as earphones, there is a gap for design and performance evaluation of such binaural systems, especially with minimal or indeed a single channel per ear for practical usage. In this paper, we explore the viability of the concept using public dataset, with a new CNN structure with modified regressed outputs proposed as a way to maximize SSVEP classification performance. The paper is organized as follows. In Methods, the dataset, the experimental setup, the proposed CNN structure, and signal processing strategies for binaural processing are described. Results considering both subject-dependent and subject-independent training methods are presented in Results, with discussion including limitations and future works in Discussion. The conclusion is given in Conclusion.

## Methods

### Dataset and Data Processing

**Figure 1:**
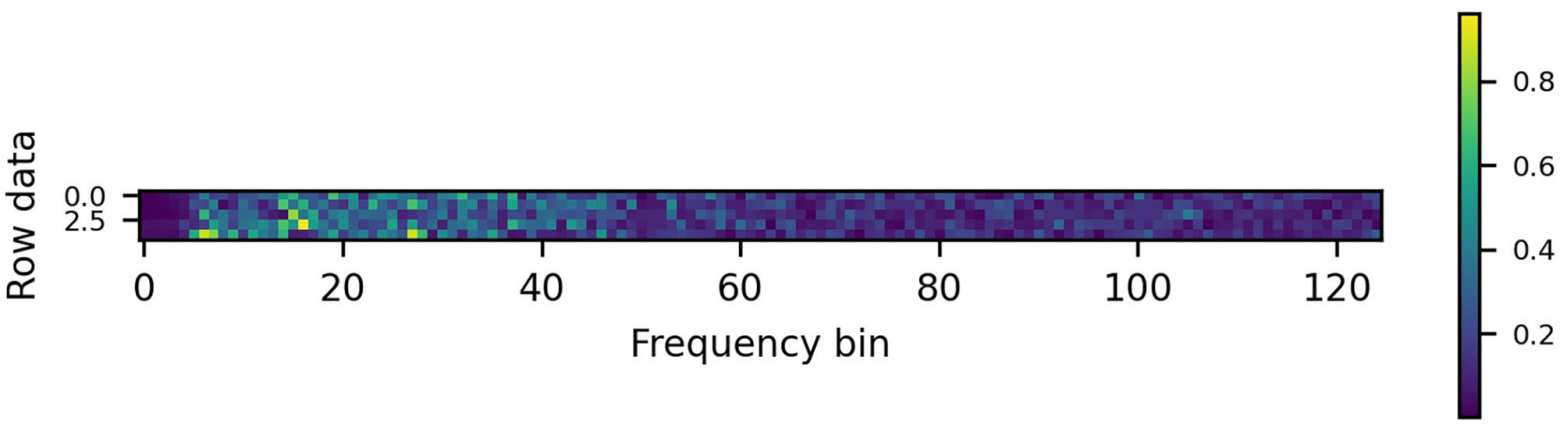
Example of a 125 × 5 spectrogram created from EEG.

The public dataset used (Wang Y. et al., 2017) contains EEG records obtained from 35 subjects, eight of which were experienced BCI users while 27 subjects did not have any prior experience in using BCIs. It was originally used to evaluate a virtual keyboard consisting of a computer display showing 40 visual flickers corresponding to different letters. The dataset has since been used in other literature such as Bassi et al. (2021). The data were recorded with a 64-channel EEG in 40 different stimulation frequencies, ranging from 8 to 15.8 Hz, with an interval of 0.2 Hz. Each subject observed the stimuli in six blocks of 40 trials, one for each frequency. The data were down sampling to 250 Hz. For each label (trial), the data length was 6 s, each with a 5-s valid data during the period from 2 to 6 s (1250 time samples). In this work, the data were band-passed with passband frequency of 5–125 Hz which were then transformed using a 250-point fast Fourier transform (FFT). Each of the 125 points representing fs/2 was then used to form each image row. The data were reshaped into spectrograms, each with the size of 125 × 5 for a 5s window length. An example of the spectrogram generated is shown in Figure 1. For the window lengths of 4, 3, 2, 1s considered in this work, the images created had the sizes of 125 × 4, 125 × 3, 125 × 2, and 125 × 1, respectively.

### Proposed CNN Structure

**Figure 2:**
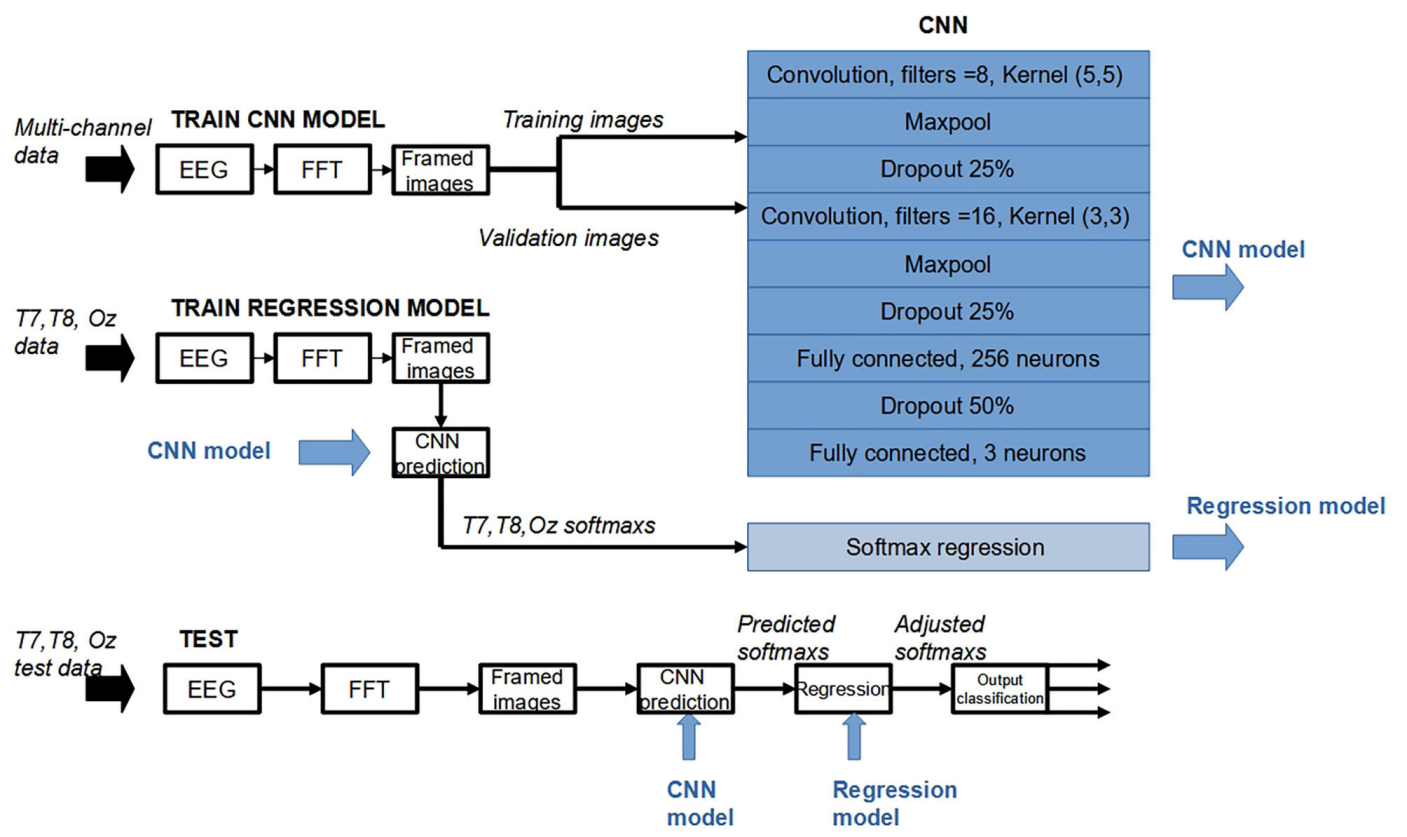
Proposed system, with CNN structure.

The deep-learning structure investigated (Figure 2) had the input layer with 2d convolution layer with (5,5) feature detector (Kernel) and a ReLu activation. The input layer was followed by a Max Pooling layer. A Dropout was added to the network as a regularization technique to prevent overfitting. Another 2d convolution layer with (3, 3) kernel, followed by another Max Pooling and Dropout layers, was next. It was then flattened to form 256 neurons fully connected layer. After another Dropout, the network was flattered to form the output layer with three neurons, each corresponded to a predicted class. A softmax function was applied to each of the three nodes in the output layer. The general form of a softmax function is given by the following equation:


(1) $$\begin{array}{l} f_{j}\left(z\right)=\frac{e^{z_{j}}}{\sum_{k=1}^{K}e^{z_{k}}},j=1,......,K \end{array}$$

*Z* is the input vector to the softmax function, made up of *K* elements (i.e., the number of classes). *z*_*j*_ is the *j*th element of *Z*. The function takes a real-valued input vector *z* and maps it to a vector of real values in the range (0, 1). Accordingly, in normal operation, an input signal can be predicted to belong to a class associated with the output whose softmax value is highest, compared to all outputs of the output layer.

**Figure 3:**
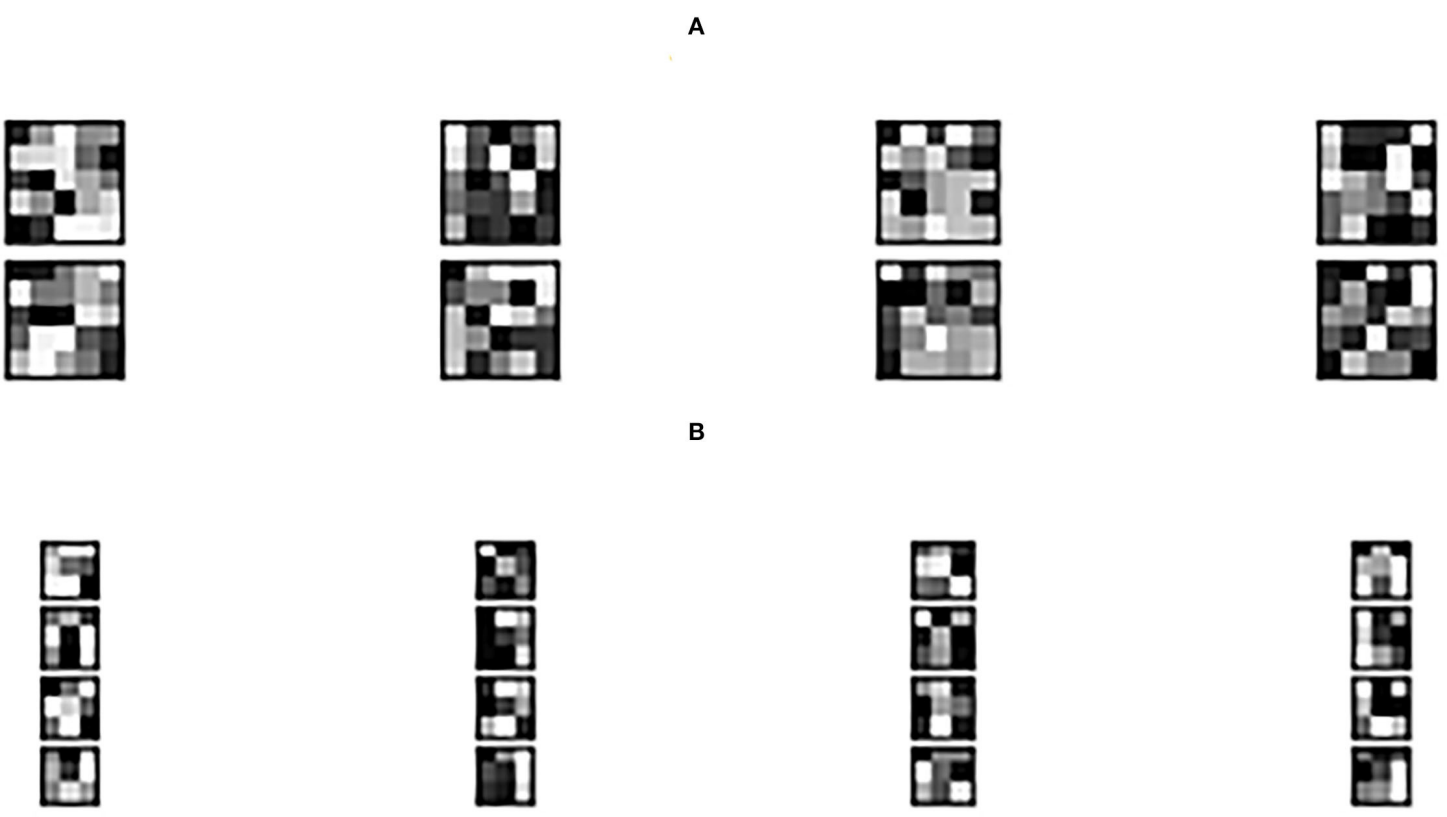
Filters: **(A)** 5 × 5 filters and **(B)** 3 × 3 filters.

**Figure 4:**
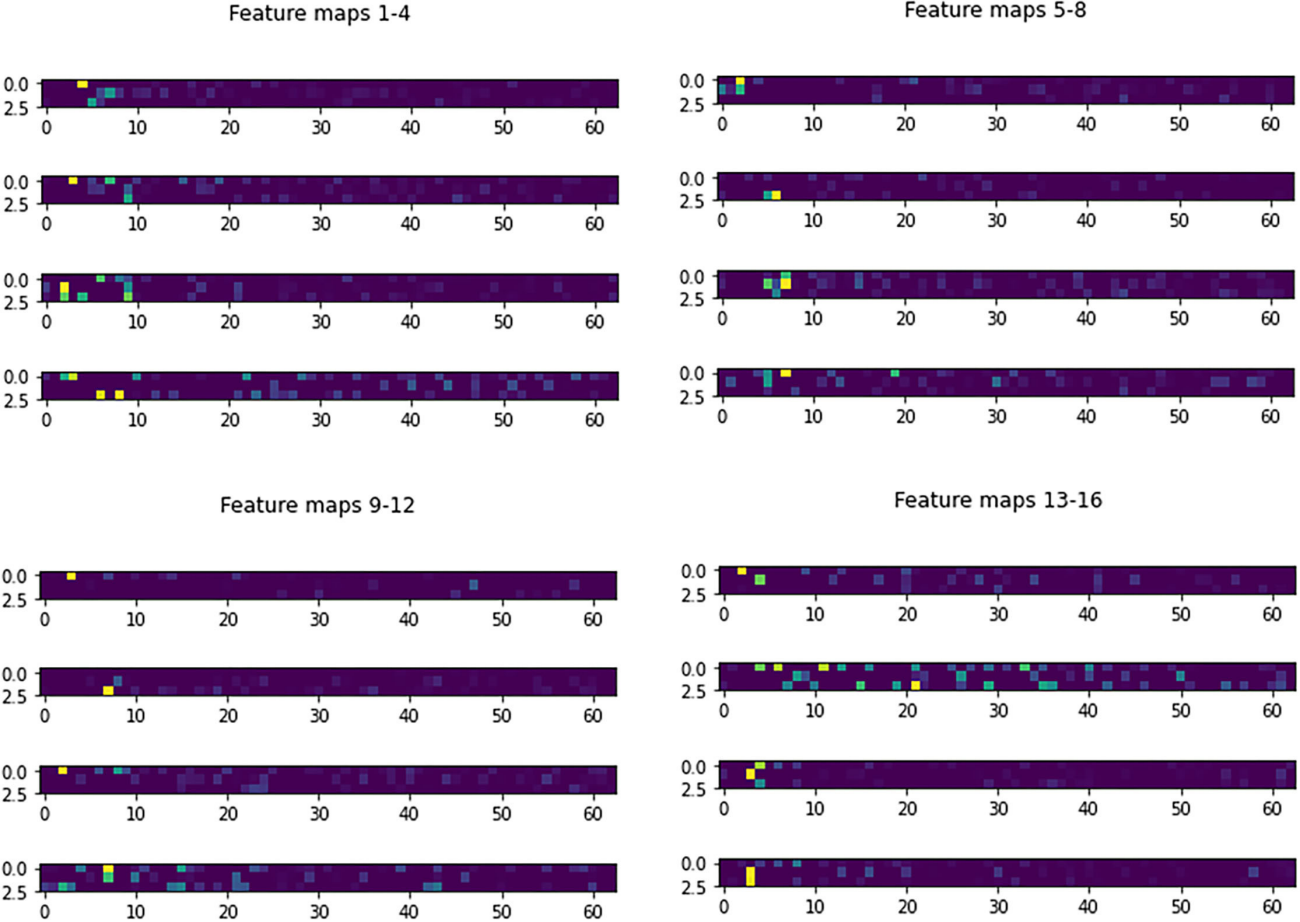
Example feature maps.

Figure 3 shows the learned 3 × 3 and 5 × 5 filters. The eight 5 × 5 filters corresponding to the first convolutional layer are shown in Figure 3A, while the sixteen 3 × 3 filters from the second convolutional layer are shown in Figure 3B. The dark squares indicate small or inhibitory weights and the light squares represent large or excitatory weights. Figure 4 shows example feature maps, which internally capture the result of applying the filters at the second convolutional layer. Intuitively, it can be seen that the system looks for different kinds of features, for example, in feature maps 6–8 the focus seems to be more on the strong signals centered around the stimulus frequency, whereas in feature map 14 the focus seems to be on the wideband noise.

For training parameters, the learning rate was set at 0.001, with the batch size 64 and the dropout rate of 0.5. The optimization algorithm was Adam, with the cross-entropy function used as the loss function (Roy et al., 2019). The stopping criteria for training CNN were number of iterations or epochs above 100.

### T7/T8 Regression

In this work, to support the binaural approach, the CNN structure was further modified after the output layer. For each softmax node, during validation session, linear regression was performed. The regression model used softmax value obtained when EEG input was from Oz as the target, and softmax reading from classification results when using T7 and T8 EEGs were used as inputs. During testing stage, the softmax outputs given EEG inputs from T7 and T8 were used as inputs in the regression model to generate “re-estimated softmaxs” (i.e., softmaxs modeled after those given Oz input). These re-estimated softmaxs were then used to predict the classes in a normal way, that is, for each sample, the maximum softmax predicted the class that was associated with that softmax node.

The CNN was implemented in Keras (https://keras.io/) and tensorflow framework (https://www.tensorflow.org/). Data were prepared using MATLAB (Mathswork Inc.).

### Experiments (Evaluation)

The proposed structure was used to train the data from the dataset to train to classify three target frequencies that were 8, 11, and 14 Hz, with window length varying from 1 to 5s. With the 3 Hz gaps, the frequencies were picked to minimize the chance of misclassification of adjacent stimulating frequencies due to noises. Performance was evaluated in terms of the accuracy and ITR (in bit/min). ITR (Nakanishi et al., 2015) stands for information transfer rate and is governed by the following equation:


(2) $$\begin{array}{l} ITR=\left(\log_{2}N+P\log_{2}P+\left(1-P\right)\log_{2}\left[\frac{1-P}{N-1}\right]\right)\times \left(\frac{60}{T}\right) \end{array}$$

where *P* is the classification accuracy, *N* is the number of stimuli, and *T* is the stimulation time including the shifting period. Here, the gaze shifting time was set at 0.55s according to simulated online performance as in the previous studies (Yin et al., 2013; Xu et al., 2014).

Two training schemes were considered.

a) Subject-dependent training
  Data were from nine channels (Oz, P7, P8, PO7, PO8, TP7, TP8, T7, and T8), with the EEGs of all 35 subjects used. The Oz channel was picked as SSVEP was known to be observable at the visual cortex (Herrmann, 2001; Han et al., 2018). This study looked for an ear-EEG application, so T7 and T8, the channels closest to the ears available, were also included. And since ERP was relatively localized with respect to brain areas, it was anticipated that by picking P7, P8, PO7, PO8, TP7, and TP8 that were adjacent channels along the line of T7-O-T8, these channels would retain similar important signal characteristics while providing appropriate additional amount of uncertainty (noise) for the classifier to generalize better. Each sample data, that is, the spectrogram, were constructed from EEG data recorded at one of the nine channels. They were randomly put into a sequence. About 90% of data was for training, and the remaining 10% of T7, T8, and Oz was used for testing with 5-fold cross-validation. By using only information from either Oz, T7, or T8 electrodes, we thus simulated a single-channel BCI.
b) Subject-independent training
  Data from the same nine channels were randomly divided into five groups, each with seven user data. Each group was tested against model trained using data from the other four groups. Results were averaged across the five test groups.

## Results

**Figure 5:**
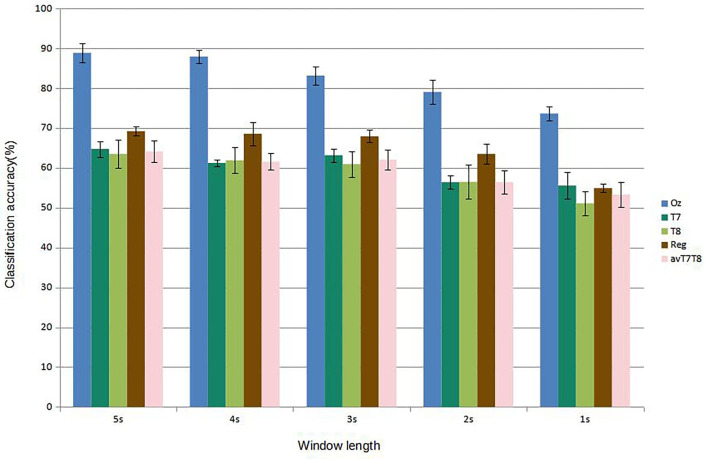
Comparison of mean classification accuracies (subject-dependent training). Error bars indicate the standard errors across the participants.

**Figure 6:**
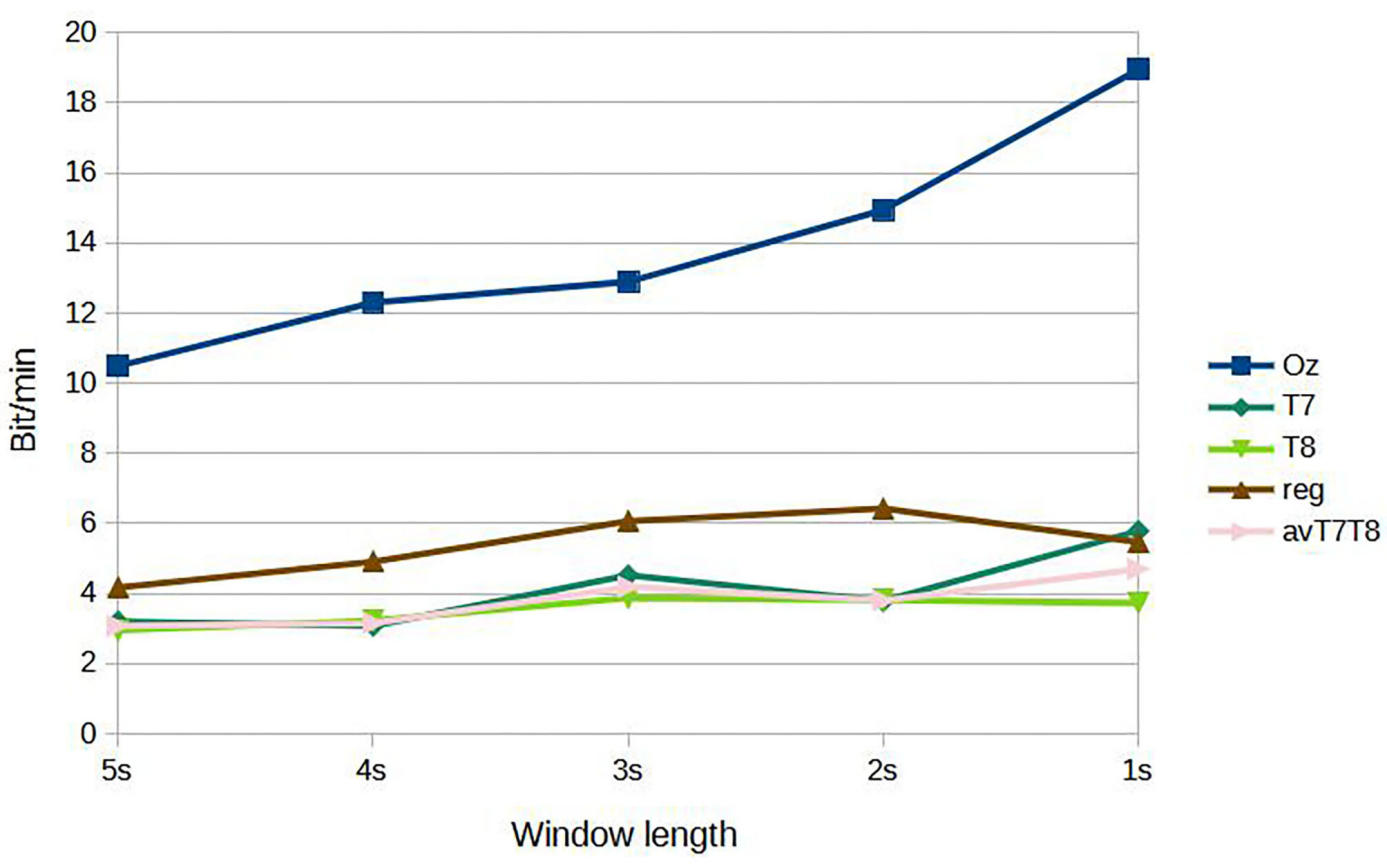
ITR, subject-dependent training.

Figure 5 shows results of subject-dependent training. It can be seen that the accuracy was best achieved by classifying signal measured at Oz, at 88.89% given the window length of 5s. The accuracy decreased to 73.65% at 1s window length. For 2s, considered a benchmark length for SSVEP accuracy measurement in many works, measuring SSVEP at Oz with the proposed CNN achieved around 79.05 %. The performance dropped considerably with classifying using either T7 or T8 data. The best accuracy was around 64.76 % at 5s window length (T7) and dropping to as low as 51.11 % with 1s window (T8). But with the regression scheme proposed, we can see that the accuracies were up for results corresponded to all the window lengths, with 69.21 % accuracy given 5s window. At 2s window, the regressed accuracy was 63.49%. The increased in accuracy with respect to the average value of T7 and T8 (avT7T8) was found to be up to 12.47%. Compared to avT7T8, the improvements were found to be significant for 2s, 3s, and 4s window lengths (P-values 0.0151, 0.0179, and 0.0160, respectively). In terms of ITR, the results given different window lengths were as in Figure 6. It can be seen that Oz achieved the rate of 18.95 bit/min with 1s window, whereas regressed binaural best was 6.42 bit/min at 2s window compared to 4.70 bit/min from T7/T8 average.

**Figure 7:**
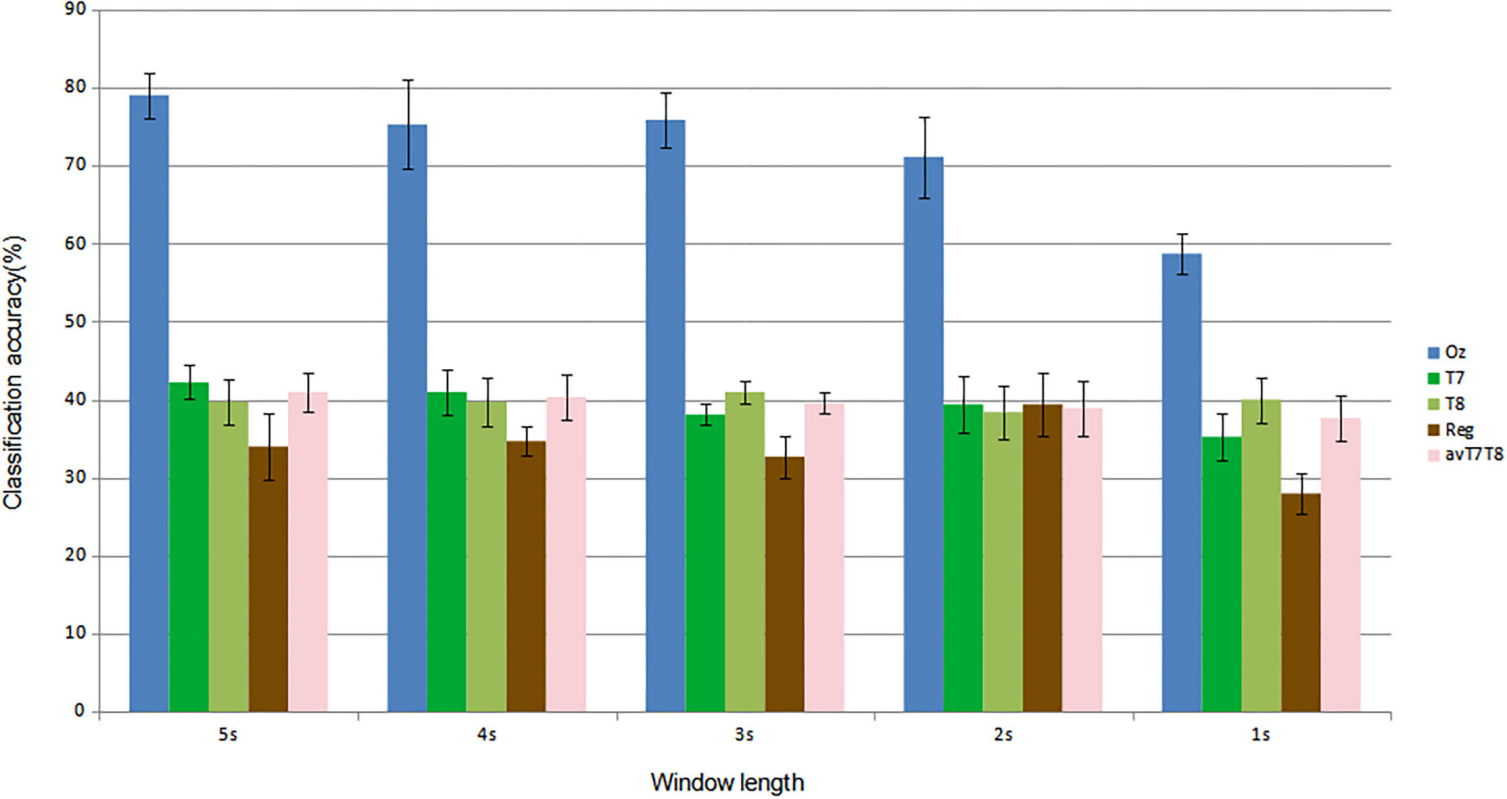
Comparison of mean classification accuracies (subject-independent training). Error bars indicate the standard errors across the participants.

**Figure 8:**
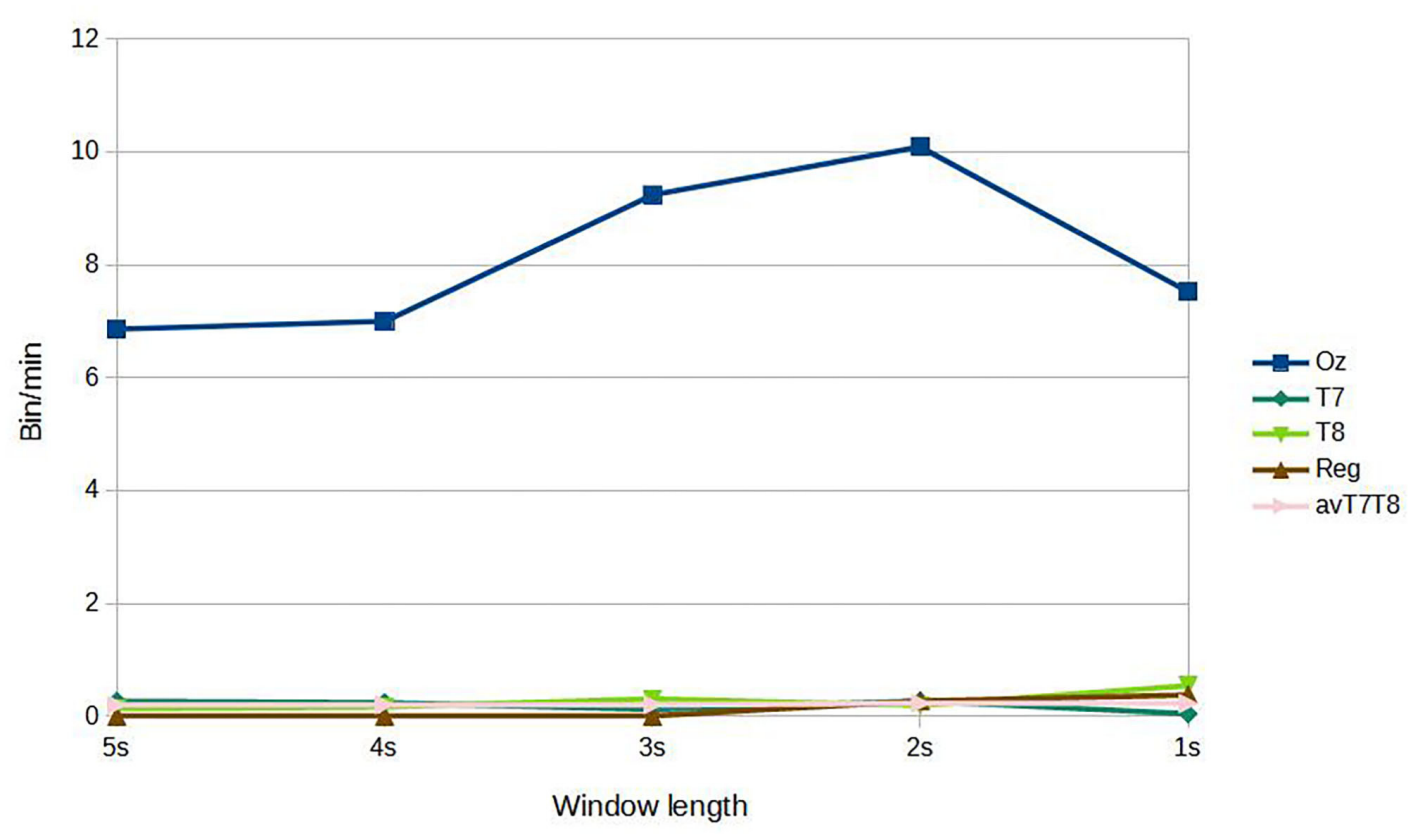
ITR, subject-independent training.

**Figure 9:**
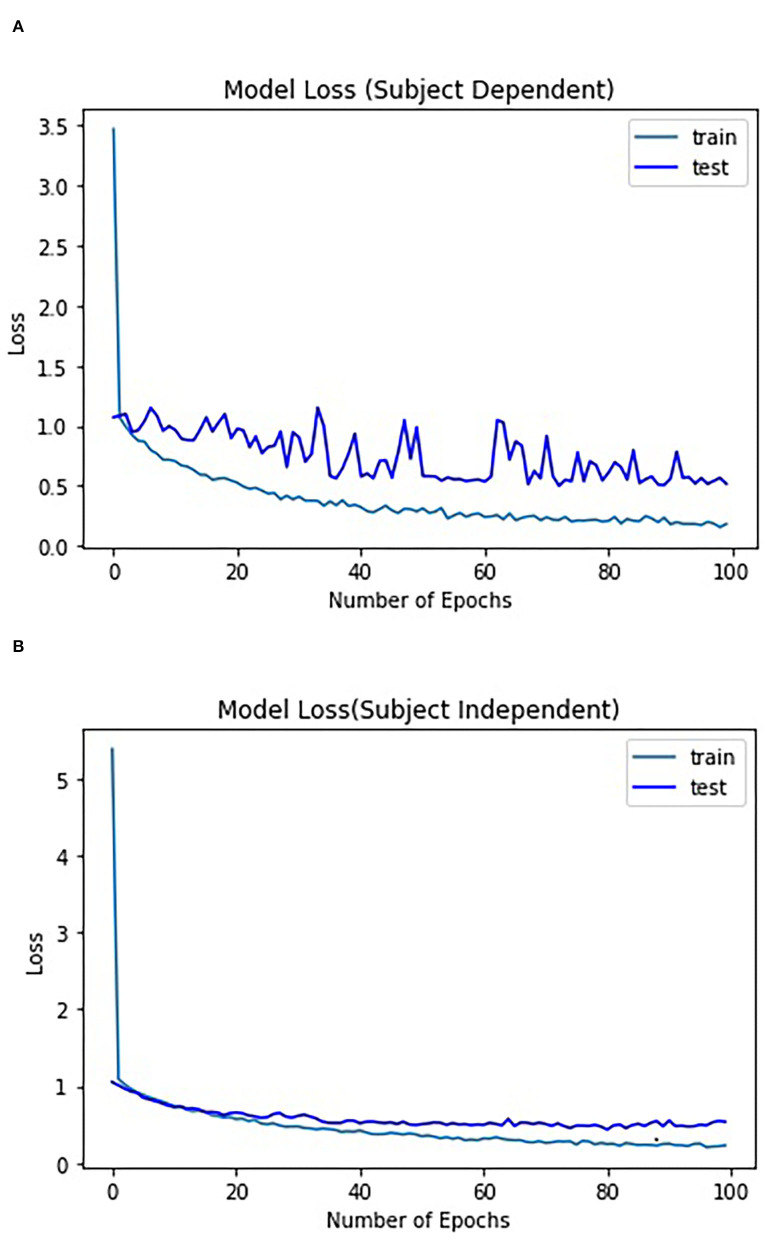
Model loss: **(A)** subject-dependent and **(B)** subject-independent.

**Figure 10:**
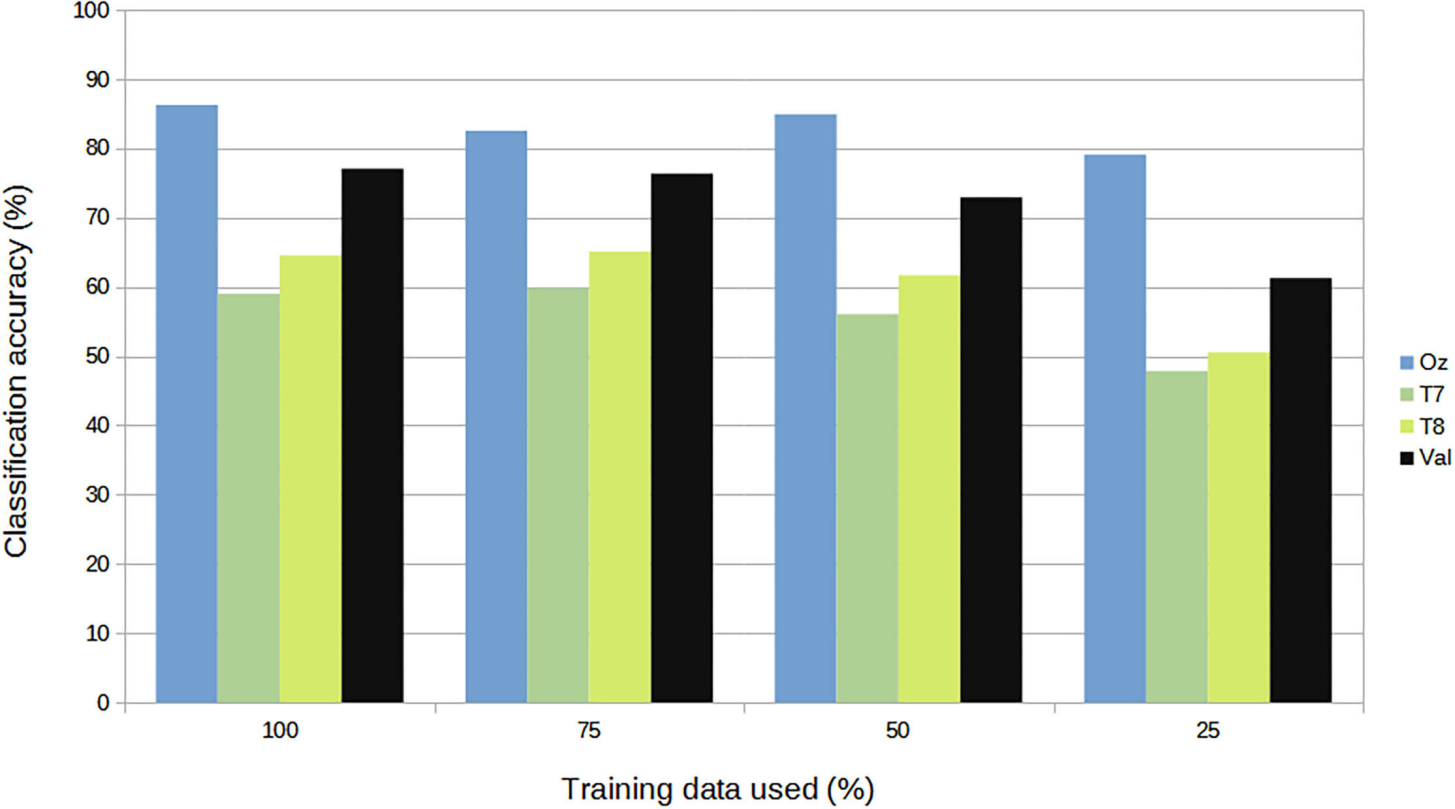
Size of dataset vs. accuracy (subject-dependent).

**Figure 11:**
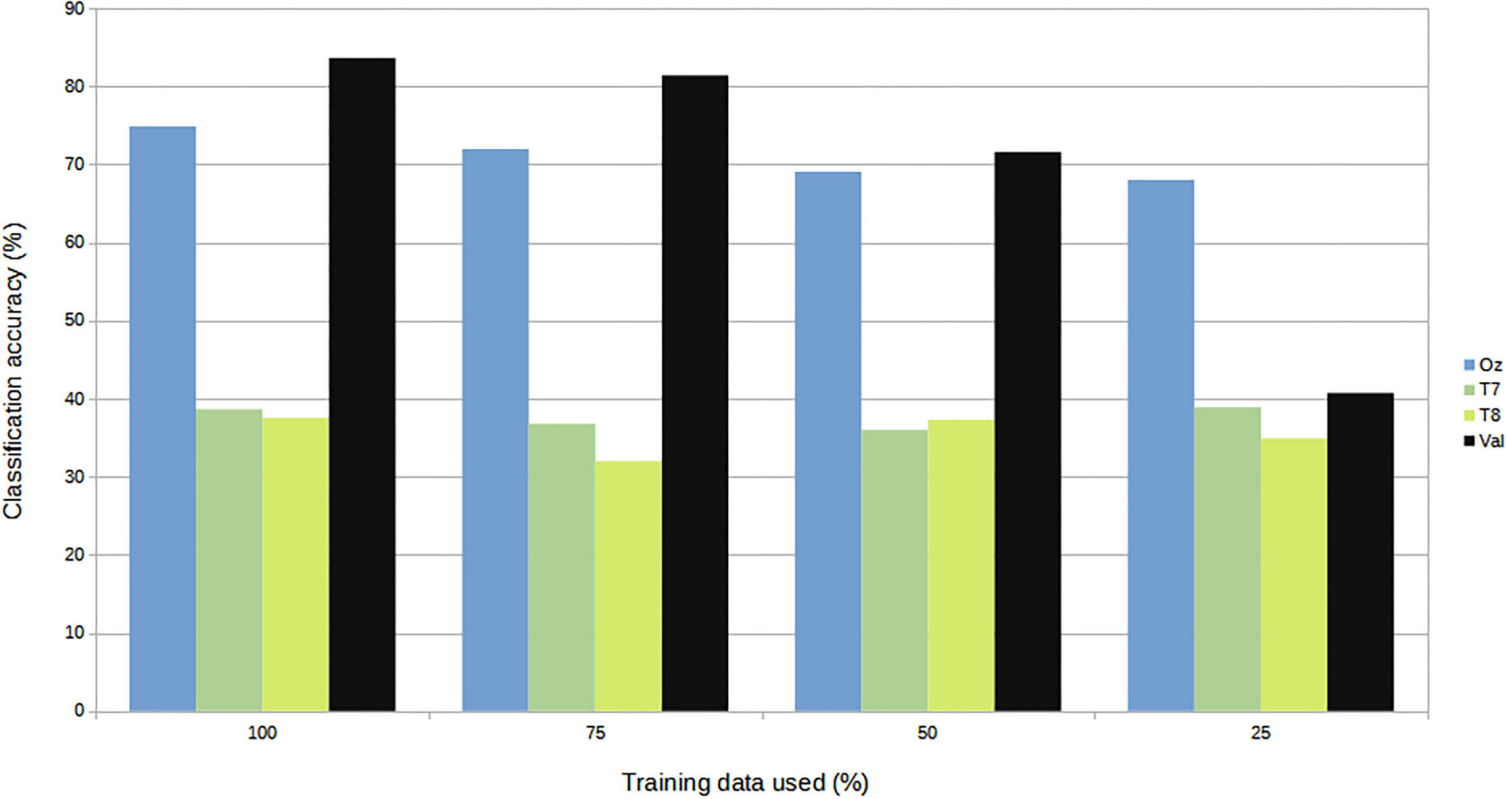
Size of dataset vs. accuracy (subject-independent).

For subject-independent training, Figures 7, 8 show accuracy and ITR results, respectively. It is clear that the performance became considerably worse than subject-dependent training in all measures. Measuring at Oz, the accuracy dropped ~10 to 79.03% at 5s window and 71.11% at 2s, whereas T7 or T8 results only achieved around 40 % regardless of the window length. The binaural regression applied also did not show improvement in this case. One possible explanation could be that subject-independent training actually made the classification problem much more difficult for neural networks, as it increased overfitting tendency (i.e., it does not generalize well). A model is said to be overfit if it is over trained on the data such that it also learns the noise from it. An overfit model learns every example so precisely that it misclassifies an unseen/new example. Usually for a model that is overfit, we see a good training set score and a poor test/validation score. Consider Figure 9 which shows typical model loss plots that were obtained from the subject-dependent and subject-independent training, respectively. We can observe a small sign of an overfitting trend in both cases, as seen from the train and test curves. This is understandable considering the relatively compact-sized dataset that we used. Deeper architectures are known to be more prone to overfitting on relatively small datasets (Goodfellow et al., 2016). Figures 10, 11 show the effect on the loss model when the size of training dataset was further reduced by 25, 50, and 75%, respectively. It can be seen that the accuracies of both validation and test models decreased further, confirming the negative effect of smaller dataset size on the accuracy. It seems, however, that the overfitting problem was amplified in the subject-independent case. As user's data were not included in training, this affected the quality of the model, resulting in a drop of performance in terms of classifying accuracy of test data. Essentially, we can consider the model not to be well generalized, as we have seen in Figure 5 compared to Figure 7. In terms of ITR (Figure 8), training with data from Oz gave best result at 10 bit/min while the rest all managed <1 bit/min ITR for all window lengths.

## Discussion

### Main Findings

**Figure 12:**
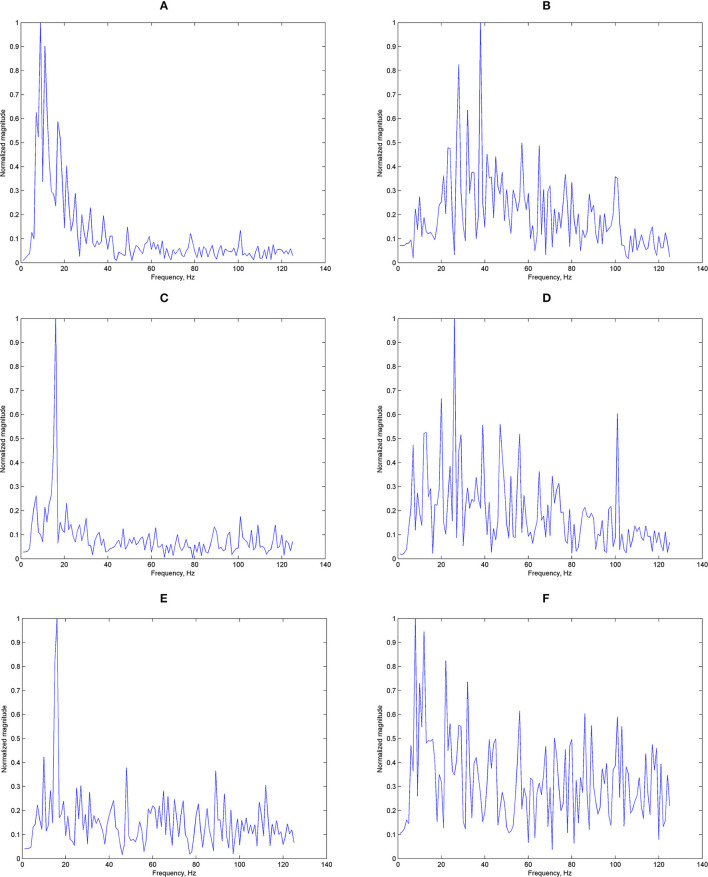
Examples of magnitude responses: **(A)** magnitude response measured at Oz, 8 Hz stimuli, **(B)** magnitude response measured at T7, 8 Hz stimuli, **(C)** magnitude response measured at Oz, 11 Hz stimuli, **(D)** magnitude response measured at T7, 11 Hz stimuli, **(E)** magnitude response measured at Oz, 14 Hz stimuli, and **(F)** magnitude response measured at T7, 14 Hz stimuli.

**Figure 13:**
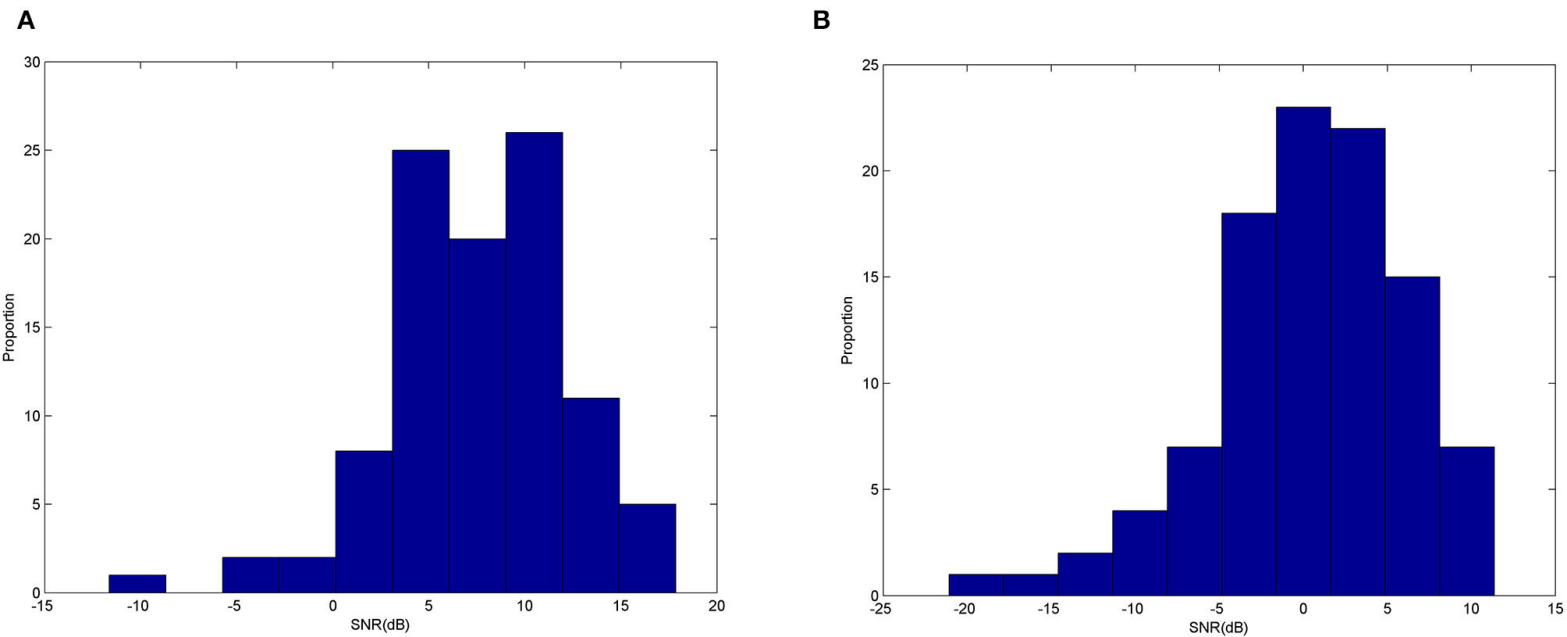
Narrowband SNRs: **(A)** as measured from Oz **(B)** as measured from T7.

We have shown the potential use for ear-EEG SSVEP in a binaural format, targeting especially single-channel earpieces which offer a practical format for real-life applications. It can be seen that generally, the accuracy got worse when classifying using signals around the ears (from either T7 or T8) compared to Oz measured at the occipital area. Figure 12 shows examples of typical Oz and T7 magnitude responses given the three stimulus frequencies. Evidently, it can already be seen that there seems to be difference in terms of the signal quality. Figure 13 shows histograms of narrowband SNR (measured at Oz and T7) from 100 randomly chosen 1s sequences. The SNRs were calculated using the following equation:


(3) $$\begin{array}{l} SNR=20\log_{10}\frac{2K.y\left(f\right)}{\sum_{k=1}^{K}\left[y\left(f-\Delta f.k\right)+y\left(f+\Delta f.k\right)\right]} \end{array}$$

*y(f)* is magnitude at the stimulus frequency, *K* is half the number of adjacent bands, and Δ*f* is the frequency step. The mean SNR from EEG measured at Oz (Figure 13A) was calculated to be 7.54 dB, much larger than 0.35 dB measured at T7 (Figure 13B). This level of difference is in line with finding from Looney et al. (2014b).

To address this shortcoming, we have shown that using the proposed CNN with group training, we could improve the ear-measured results by 12.47%, closing the gap toward the level obtained when using signal from Oz for classification. This was achieved with a practical range of 2s window length. In terms of the training strategy, subject-dependent training was also found to be better than subject-independent training. This may be due to the longer training set overall, and the variety of data led to the model being able to generalize better. Considering the computational time, we also believe the proposed method is capable of supporting real-time BCI applications. For example, to process a batch of 100 images with pre-trained model only required around 0.2 s based on a system with Core i7 CPU and RTX2070 GPU. The main latency would still be determined mainly by the window length selected, similar to other implementation methods.

Compared to other literatures, most have focused on measuring the SSVEP signal from the occipital area. Nakanishi et al. (2015) showed that with 8-channel EEG measurement taken from the occipital and posterior areas, the proposed design achieved more than 90% accuracy (depending on the window length) with up to 91.68 bits/min ITR achievable for simulated online BCI compared more favorably to results from the more conventional CCA approach (50% accuracy approximately at 2s window, 50.4 bits/min ITR). In Ravi et al. (2020), with 6-channel recordings, CCA achieved 62–69% accuracy with short window length of 1s. It also studied the effects of user-independent (UI) and user-dependent (UD) trainings, with UD-based training methods consistently outperforming the UI methods, which agree with our finding. Extremely fast design was proposed in Nakanishi et al. (2018). With 40 classification targets, 89.83% accuracy and 198.67 bits/min ITR for free spelling task were reported. Albeit offering a high ITR, the complicated interface associated with having many stimuli (40 stimuli in this case) could be a challenge to the user and may not be suitable for certain applications, for example, mobile-based ones. It is noted also that the design also required online training. In all, it should be re-emphasized that all these multi-channel systems require controlled environment and delicate setting up arrangements.

For more mobile solutions, one potential answer is to use a single-channel measuring at Oz format. Nguyen and Chung (2019) designed their own EEG amplifier and in an *N* = 8 trial achieved the accuracy of 99.2 % (offline) and 97.4 % (online), with ITR of 49 bits/min when measuring at Oz. Using 1-D DNN, the system classified five targets using 2s windows. Benchmark CCA implementation was shown to achieve lower accuracy, at around 80%. The system also required user-dependent training scheme. Bassi et al. (2021) suggested a very short window time of 0.5s. Using CNN with transfer learning to classify two targets, they achieved 82.2% accuracy. It is interesting to note that the reported system was tested on the same dataset and achieved similar results to the Oz reference measurement here (0.5s 2 classes vs 2s 3 classes).

For ear-EEG, most took the multi-channel approach using the cEEGed platform (Debener et al., 2015). With up to 18 channels reading from two ears, Zhu et al. (2021) achieved 81–84% accuracy with 1s window length, slightly less than >90% accuracy from scalp reading. In the work, a CCA-based SSVEP measuring from ears provided as reference was found to be only around 40–50% accurate. The dataset used was actually from Kwak and Lee (2020), which achieved 80–90% accuracy at 6s window, or around 60–70% at 2s window. Kwak and Lee (2020) classified three classes with 91/90/86% accuracies for single session, session-to-session transfer, and subject-transfer decoding, respectively, using 6s window. For online implantation, 18.07 bits/min ITR was achieved with 78.79 % accuracy. For 2s window, the accuracy was reduced to <60%, with the error correct framework improved it to 78.79%. Other than the cEEGed, Wang et al. (2015) used 6x2 channels with bio semi amplifier plus custom ears, achieved 78.75% (offline) and 87.44% (online) accuracies. The window length was 4s, achieving the ITR of 15.71 s. The performance was comparable to others or to this work, but the number of subjects used was rather small at only 2. In Lan et al. (2021), performance measured at the ear areas was compared to those measured around the occipital area. Using task-related component analysis (TRCA) to classify eight classes with 5s window, the measurement at either ear (three channels, FT7(8), T7(8), TP7(8)) achieved ~35–44% accuracy depending on the type of reference used. The accuracy improved to around 50–55% with six channels (three from both ears) which was <69% accuracy we achieved with the two-ear regression. Lan also showed that with only three channels at the occipital area, accuracy above 90% could be achieved.

**Figure 14:**
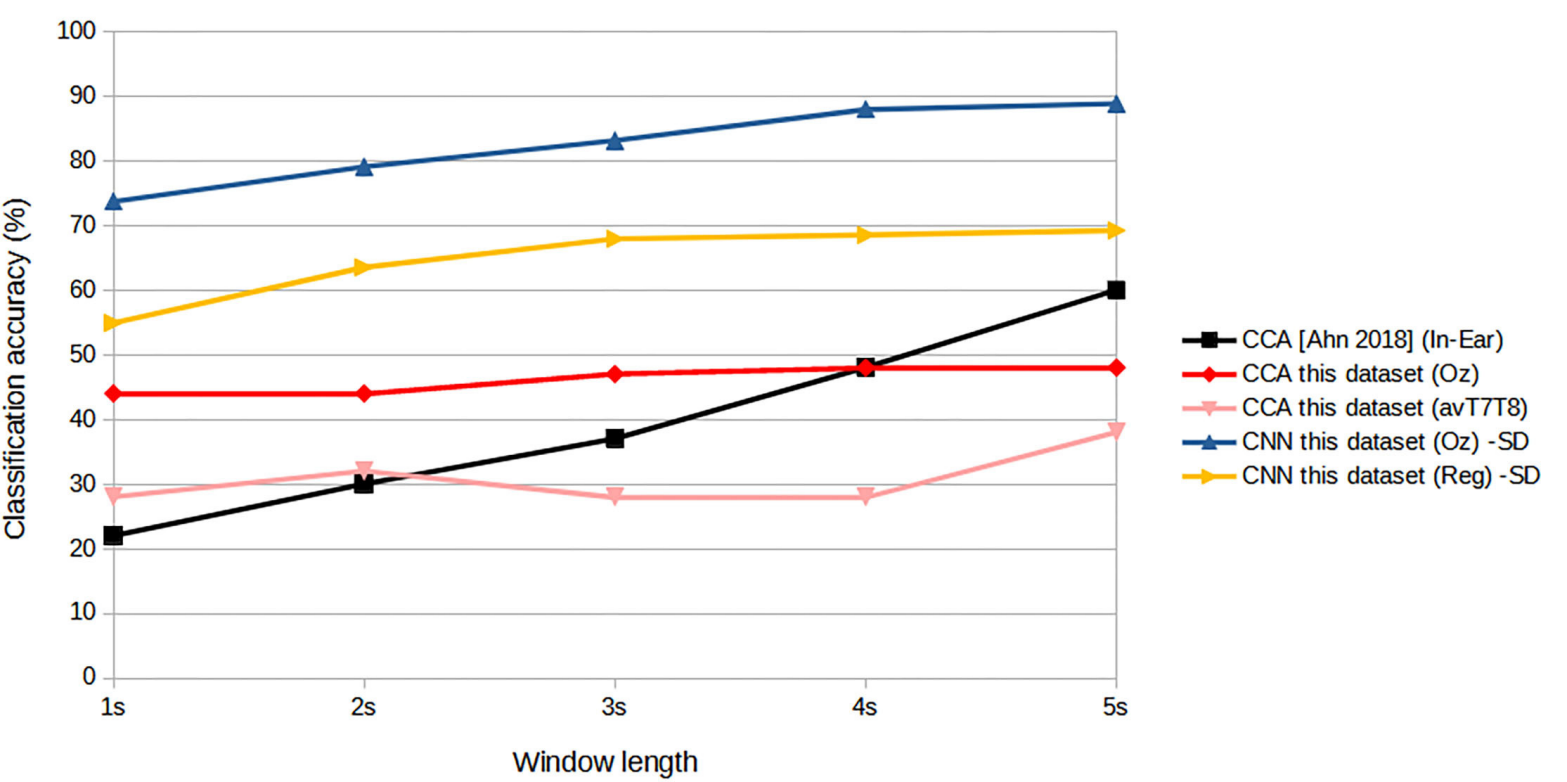
Comparison with CCA.

Consider the practicality issues with minimal channel ear-EEG, (Looney et al., 2011) Looney's original ear-EEG had two-channel in-ear electrodes. It was shown that the degree of coherence was high between the in-ear electrodes measurement and those measured from the on-scalp T7, T8 areas. The most similar in concept with performance measurement reported was Ahn et al. (2018), which was a single-channel in-ear EEG, tested on six persons. The accuracy was reported to be 79.9% with ITR 11.3 bits/min for six-target classification. This, however, was achieved with 7s window length which could be considered impractically too long for online applications, especially for controlling external devices. From the paper's graph, it can be estimated that for 2s window, the accuracy was reduced to only around 30% with ITR of 3 bits/min which is less than the performance reported here. To explore this further, we have reconstructed a CCA and measured the classification performance given the same dataset as the one we used. The results of Ahn's CCA and our CNN-based classifications are shown in Figure 14. Given CCA classification at ear area (avT7T8) for this particular dataset, it can be seen that the accuracy matches that of Ahn's in-ear work with 2s window length, but exhibits increasing gap as window length increases. CCA classification from signals at Oz improves the accuracy as expected, with better results against Ahn's work for window lengths up to 4 s. CNN-based designs were superior to all CCA designs, with the regressed T7T8 design outperforming the in-ear CCA counterpart.

The difference is summarized in Table 1. Carvalho et.al.'s work (Carvalho et al., 2021) which used the same dataset is also included for comparison.

**Table 1:** SSVEP performance comparison.

**Work**	**Tech**	**EEG**	**CH**	**Position**	**Subject N [dataset]**	**Window length**	**No of class**	**Accuracy %**	**ITR (bit/min)**
Nakanishi et al., 2015	IT-CCA	Biosemi ActiveTwo EEG	8	Op	10 [own]	varies	12	>90 (CCA 50)	91.68 (CCA 50.4)
Ravi et al. (2020)	CCA, FBCCA, TRCCA	g.USBAmp	6	O1, O2, Oz PO3, PO4, Poz	121[own]/ 10[Nakanishi et al., 2015]	0.5s−3s	7	CCA 62–69 (1s)	N/A
Nakanishi et al. (2018)	TRCA	Neuroscan Synamps2	9	Pz, PO5, PO3, Poz, PO4, PO6, O1, Oz, O2	12[own]	0.5s offline 0.3s online	40	89.83	198.67
Nguyen and Chung (2019)	1-D CNN	Custom	1	O1-Oz pair	8[own]	2s	5	99.2 offline 97.4 online	49
Bassi et al. (2021)	DCNN/ Transfer Learning	Neuroscan Synamps2	1	Oz	35 [Wang Y. et al., 2017]	0.5s	2	82.2	N/A
Zhu et al. (2021)	EEGnet/ Ensemble Learning	CEEGrid + mBrain Train Smarting System	18 (2 × 9)	2X round-the-ear	11 [Kwak and Lee, 2020]	1s	3	81–84	N/A
Kwak and Lee (2020)	Error Correction Regression	CEEGrid + mBrain Train Smarting System	18 (2 × 9)	2X round-the-ear	11[own]	2s,4s,6s	3	6s: 91/90/86 (single ses/ ses-to-ses/ sbj-trans) 2s: <60	2s: 18.07 online (78.79% accuracy)
Wang et al. (2015)	Extended CCA	Custom mold + Biosemi ActiveTwo EEG	12 (2 × 6)	2X In-ear	2[own]	4s	4	78.75 (offline) 87.44 (online)	15.71
Ahn et al. (2018)	CCA	Custom	1	1X In-ear	6[own]	7s	6	79.9	11.03
Lan et al. (2021)	TRCA	Neuroscan Synamps2	3/6 3/6/9	FT7, T7, TP7, FT8, T8, TP8 O1, Oz, O2, PO5, PO3, POz, PO4, PO6, PZ	35 [Wang Y. et al., 2017]	5s	8	35-44% / 50-55% >90% / >97%/>97%	7 apx/11 apx 30 apx/32 apx/32 apx
Carvalho et al., 2021	MVDR-CAR	Neuroscan Synamps2	16	O1, O2, Oz, POz, Pz, PO3, PO4, PO7, PO8, P1, P2, Cz, C1, C2, CPz, FCz)	35 [Wang Y. et al., 2017]	3s	4/6	98%(96% CCA) /98% (83% CCA)	N/A
Israsena and Pan-ngum	CNN/ Binaural Regression	Neuroscan Synamps2	2	T7,T8	35 [Wang Y. et al., 2017]	2s	3	69.21	6.42

In summary, we see or confirm the following trends:

- User-dependent training for better performance.
- Machine learning over conventional CCA.
- Performance drops from measuring at Oz to ear areas.
- Better performance in binaural ear-EEG from our proposed design compared to designs with similar specs.

### Limitations and Future Work

In this work, we studied the effect of SSVEP classification from EEG measured around the ear using data collected from T7 and T8 areas. Although research has shown T7 and T8 characteristics to match those of in-ear measurements, actual measurements from in-ear positions will help further confirm the results discussed here. Also, as we looked for minimal channel for practical/mobility, another factor to consider in these conditions is ambulatory. Works such as Lee and Lee (2020) have already shown that ML approach is more robust against a number of ambulatory conditions. Dataset also matters, as (Nakanishi et al., 2015; Ravi et al., 2020) already shown effects of different datasets used on performance evaluation. Here, we use dataset from Wang Y. et al. (2017) which is rather compact. Although this helps evaluate whether the system is robust with small dataset, which may be beneficial in real-world applications, longer training data could potentially improve accuracy further as deeper architectures are more prone to overfitting on relatively small datasets (Goodfellow et al., 2016). Lastly, the group training approach requires access to subject's data. This individualized data may be acquired during calibration process, and this is our recommended approach.

## Conclusion

This paper discusses a machine learning approach for measuring SSVEP at both ears with minimal channel. We propose a new CNN-based approach that was coupled with regressed softmax output classification to improve accuracy. With the proposed structure using group training approach, a 69.21% accuracy was achievable. An ITR of 6.42 bit/min given 63.49 % accuracy was recorded while only monitoring data from T7 and T8, representing a 12.47% improvement from a single ear implementation and illustrating the potential approach to enhance performance for practical implementation of wearable EEG.

## Data Availability Statement

Publicly available datasets were analyzed in this study. This data can be found here: Tsinghua University Brain-Computer Interface (BCI) Research Group database, http://bci.med.tsinghua.edu.cn/.

## Author Contributions

PI and SP-N conceived and designed the study, supervised the study, and reviewed and edited the manuscript. PI performed the analysis and wrote the manuscript. Both authors approved the final manuscript.

## Funding

This work was supported by the National Metal and Materials Technology Center (MTEC) through the NSTDA Frontier in Exoskeleton program.

## Conflict of Interest

The authors declare that the research was conducted in the absence of any commercial or financial relationships that could be construed as a potential conflict of interest.

## Publisher's Note

All claims expressed in this article are solely those of the authors and do not necessarily represent those of their affiliated organizations, or those of the publisher, the editors and the reviewers. Any product that may be evaluated in this article, or claim that may be made by its manufacturer, is not guaranteed or endorsed by the publisher.

## References

[B1] Abdel-Hamid O. Mohamed A. R. Jiang H. Deng L. Penn G. Yu D. (2014). Convolutional neural networks for speech recognition. IEEE/ACM Trans. Audio Speech Lang. Process. 22, 1533–1545. 10.1109/TASLP.2014.233973635327924

[B2] Ahn J. Ku Y. Kim D. Y. Sohn J. Kim J. H. Kim H. C. (2018). Wearable in-the-ear EEG system for SSVEP-based brain–computer interface. Electron. Lett. 54, 413–414. 10.1049/el.2017.3970

[B3] Bassi P. Rampazzo W. Attux R. (2021). Transfer Learning and SpecAugment applied to SSVEP Based BCI Classification. Biomed. Signal Process. Control 67, 102542. 10.1016/j.bspc.2021.102542

[B4] Bin G. Gao X. Wang Y. Hong B. Gao S. (2009). Research frontier: VEP-based brain-computer interface: time, frequency, and code modulations. IEEE Comput. Intell. Mag. 4, 22–26. 10.1109/MCI.2009.934562

[B5] Bin G. Gao X. Wang Y. Hong B. Gao S. (2011). A high-speed BCI based on code modulation VEP. J. Neural Eng. 8, 025015. 10.1088/1741-2560/8/2/025015 21436527

[B6] Bleichner M. G. Debener S. (2017). Concealed, unobtrusive ear-centered EEG acquisition: cEEGrids for transparent EEG. Front. Hum. Neurosci. 11, 163. 10.3389/fnhum.2017.00163 28439233 PMC5383730

[B7] Bleichner M. G. Mirkovic B. Debener S. (2016). Identifying auditory attention with ear-EEG: cEEGrid versus high-density cap-EEG comparison. J. Neural Eng. 13, 066004. 10.1088/1741-2560/13/6/066004 27705963

[B8] Carvalho S. N. Vargas G. V. da Silva Costa T. B. de Arruda Leite H. M. Coradine L. Boccato L.. (2021). Space-time filter for SSVEP brain-computer interface based on the minimum variance distortionless response. Med. Biol. Eng. Comput. 59, 1133–1150. 10.1007/s11517-021-02345-7 33909252

[B9] Chang L. Wang R. Zhang Y. (2022). Decoding SSVEP patterns from EEG via multivariate variational mode decomposition-informed canonical correlation analysis. Biomed. Signal Process. Control 71, 103209. 10.1016/j.bspc.2021.103209

[B10] Chen X. Chen Z. Gao S. Gao X. (2014a). A high-ITR SSVEP based BCI speller. Brain Comput. Interfaces 1, 181–191. 10.1080/2326263X.2014.944469

[B11] Chen X. Wang Y. Nakanishi M. Jung T. P. Gao X. (2014b). Hybrid frequency and phase coding for a high-speed SSVEP-based BCI speller. Ann. Int. Conf. IEEE Eng. Med. Biol. Soc. 2014, 3993–3996. 10.1109/EMBC.2014.6944499 25570867

[B12] Cheng M. Gao X. Gao S. Xu D. (2002). Design and implementation of a brain-computer interface with high transfer rates. IEEE Trans. Biomed. Eng. 49, 1181–1186. 10.1109/TBME.2002.803536 12374343

[B13] Christensen C. B. Harte J. M. Lunner T. Kidmose P. (2018). Ear- EEG-based objective hearing threshold estimation evaluated on normal hearing subjects. IEEE Trans. Biomed. Eng. 65, 1026–1034. 10.1109/TBME.2017.2737700 28796603

[B14] Debener S. Emkes R. De Vos M. Bleichner M. (2015). Unobtrusive ambulatory EEG using a smartphone and flexible printed electrodes around the ear. Sci. Rep. 5, 16743. 10.1038/srep16743 26572314 PMC4648079

[B15] Farwell L. A. Donchin E. (1988). Talking off the top of your head: Toward a mental prosthesis utilizing event-related brain potentials. Electroencephalogr. Clin. Neurophysiol. 70, 510–523. 10.1016/0013-4694(88)90149-6 2461285

[B16] Gao S. Wang Y. Gao X. Hong B. (2014). Visual and auditory brain computer interfaces. IEEE Trans. Biomed. Eng. 61, 1436–1447. 10.1109/TBME.2014.2300164 24759277

[B17] Goodfellow I. Bengio Y. Courville A. Bengio Y. (2016). Deep Learning, vol. 1. Cambridge: MIT Press.

[B18] Goverdovsky V. von Rosenberg W. Nakamura T. Looney D. Sharp D. J. Papavassiliou C.. (2017). Hearables: multimodal physiological in-earsensing. Sci. Rep. 7, 6948. 10.1038/s41598-017-06925-2 28761162 PMC5537365

[B19] Goverdovsky V. Looney D. Kidmose P. Mandic D. P. (2016). In-ear EEG from viscoelastic generic earpieces: Robust and unobtrusive 24/7 monitoring. IEEE Sensors J. 16, 271–277. 10.1109/JSEN.2015.2471183

[B20] Gu Y. Cleeren E. Dan J. Claes K. Van Paesschen W. Van Huffel S.. (2017). Comparison between scalp EEG and behind-the-ear EEG for development of a wearable seizure detection system for patients with focal epilepsy. Sensors. 18, 29. 10.3390/s18010029 29295522 PMC5795884

[B21] Han C. Xu G. Xie J. Chen C. Zhang S. (2018). Highly interactive brain-computer interface based on flicker-free steady-state motion visual evoked potential. Sci. Rep. 8, 5835. 10.1038/s41598-018-24008-8 29643430 PMC5895715

[B22] Herrmann C. S. (2001). Human EEG responses to 1–100 Hz flicker: Resonance phenomena in visual cortex and their potential correlation to cognitive phenomena. Exp. Brain Res. 137, 346–353. 10.1007/s002210100682 11355381

[B23] Kam J. W. Y. Griffin S. Shen A. Patel S. Hinrichs H. Heinze H. J.. (2019). Systematic comparison between a wireless EEG system with dry electrodes and a wired EEG system with wet electrodes. NeuroImage 184, 119–129. 10.1016/j.neuroimage.2018.09.012 30218769 PMC6568010

[B24] Kappel S. L. Kidmose P. (2017). High-density ear-EEG. Annu. Int. Conf. IEEE Eng. Med. Biol. Soc. 2394–2397. 10.1109/EMBC.2017.8037338 29060380

[B25] Kappel S. L. Loonney D. Mandic D. P. Kidmose P. (2014). A method for quantitative assessment of artifacts in EEG, and an empirical study of artifacts. Annu. Int. Conf. IEEE Eng. Med. Biol. Soc. 2014, 1686–1690. 10.1109/EMBC.2014.6943931 25570299

[B26] Kappel S. L. Rank M. L. Toft H. O. Andersen M. Kidmose P. (2019). Dry-contact electrode ear-EEG. IEEE Trans. Biomed. Eng. 66, 150–158. 10.1109/TBME.2018.2835778 29993415

[B27] Kidmose P. David L. Michael U. Mike L. R. Danilo P. M. (2013). A study of evoked potentials from ear-EEG. IEEE Trans. Biomed. Eng. 60, 2824–2830. 10.1109/TBME.2013.2264956 23722447

[B28] Kim D. W. Lee J. C. Park Y. M. Kim I. Y. Im C. H. (2012). Auditory brain–computer interfaces (BCIs) and their practical applications. Biomed. Eng. Lett. 2, 13–17. 10.1007/s13534-012-0051-1

[B29] Krizhevsky A. Sutskever I. Hinton G. E. (2012). “Imagenet classification with deep convolutional neural networks,” in Advances in Neural Information Processing Systems, 1097–1105.

[B30] Kwak N. S. Lee S. W. (2020). Error Correction Regression Framework for enhancing the decoding accuracies of ear-EEG brain-computer interfaces. IEEE Trans. Cybern. 50, 3654–3667. 10.1109/TCYB.2019.2924237 31295141

[B31] Kwak N. S. Müller K. R. Lee S. W. (2017). A convolutional neural network for steady state visual evoked potential classification under ambulatory environment. PLoS ONE. 12, 1–20. 10.1371/journal.pone.0172578 28225827 PMC5321422

[B32] Lan W. Yang H. Leng Y. Wang R. Iramina K. Ge S. (2021). “Effect of Channel and Reference Selection on a Non-occipital Steady-State Visual Evoked Potential-Based Brain-Computer Interface,” in 5th IEEE Information Technology, Networking, Electronic and Automation Control Conference (ITNEC), 1274–1280.

[B33] Lee J. S. Lee S. M. Byeon H. J. Hong J. S. Park K. S. Lee S. H. (2014). CNT/PDMS-based canal-typed ear electrodes for inconspicuous EEG recording. J. Neural Eng. 11, 046014. 10.1088/1741-2560/11/4/046014 24963747

[B34] Lee Y. Lee M. (2020). “Decoding Visual Responses based on Deep Neural Networks with Ear-EEG Signals,” in 8th International Winter Conference on Brain-Computer Interface (BCI), 1–6.

[B35] Lin Z. Zhang C. Wu W. Gao X. (2007). Frequency recognition based on canonical correlation analysis for SSVEP-based BCIs. IEEE Trans. Biomed. Eng. 54, 1172–1176. 10.1109/TBME.2006.889197 17549911

[B36] Looney D. Kidmose P. Mandic D. P. (2014b). “Ear-EEG: Usercentered and wearable BCI,” in Brain–Computer Interface Research: A State-of-the-Art Summary 2, eds Guger C. Allison B. Leuthardt E. C. (Cham, Switzerland: Springer), 41–50.

[B37] Looney D. Kidmose P. Morrell M. J. Mandic D. P. (2014a). “Ear- EEG: Continuous brain monitoring,” in Brain–Computer Interface Research: A State-of-the-Art Summary 3, eds. Guger C. Vaughan T. Allison B. (Cham, Switzerland: Springer), 63–71.

[B38] Looney D. Kidmose P. Park C. Ungstrup M. Rank M. R. Rosenkranz K.. (2012). The in-the-ear recording concept: User-centered and wearable brain monitoring. IEEE Pulse Mag. 3, 3242. 10.1109/MPUL.2012.2216717 23247157

[B39] Looney D. Park C. Kidmose P. Rank M. L. Ungstrup M. Rosenkranz K.. (2011). “An In-The-Ear Platform For Recording Electroencephalogram,” in Int. Conf. of the IEEE Engineering in Medicine and Biology Society (EMBC), 6882–6885. 22255920 10.1109/IEMBS.2011.6091733

[B40] Marini F. Lee C. Wagner J. Makeig S. Gola M. (2019). A comparative evaluation of signal quality between a research-grade and a wireless dry-electrode mobile EEG system. J. Neural Eng. 16, 054001. 10.1088/1741-2552/ab21f2 31096191

[B41] Mellinger J. Schalk G. Braun C. Preissl H. Rosenstiel W. Birbaumer N.. (2007). An MEG-based brain–computer interface (BCI). NeuroImage. 36, 581–593 10.1016/j.neuroimage.2007.03.019 17475511 PMC2017111

[B42] Mikkelsen K. B. Kappel S. L. Mandic D. P. Kidmose P. (2015). EEG recorded from the ear: Characterizing the ear- EEG method. Front. Neurosci. 9, 438. 10.3389/fnins.2015.00438 26635514 PMC4649040

[B43] Mirkovic B. Bleichner M. G. De Vos M. Debener S. (2016). Target speaker detection with concealed EEG around the ear. Front. Neurosci. 10, 349. 10.3389/fnins.2016.00349 27512364 PMC4961688

[B44] Nakamura T. Goverdovsky V. Mandic D. P. (2018). In-ear EEG biometrics for feasible and readily collectable real-world person authentication. IEEE Trans. Inf. Forensics Security. 13, 648–661. 10.1109/TIFS.2017.2763124

[B45] Nakanishi M. Wang Y. Chen X. Te Wang Y. Gao X. Jung T. P. (2018). Enhancing detection of SSVEPs for a high-speed brain speller using task-related component analysis. IEEE Trans. Biomed. Eng. 65, 104–112. 10.1109/TBME.2017.2694818 28436836 PMC5783827

[B46] Nakanishi M. Wang Y. Wang Y. T. Mitsukura Y. Jung T. P. (2014), A high-speed brain speller using steady-state visual evoked potentials. Int. J. Neural Syst. 24, 1450019. 10.1142/S0129065714500191 25081427

[B47] Nakanishi M. Wang Y. T. Wang Y. Jung T. P. (2015). A comparison study of canonical correlation analysis based methods for detecting steady-state visual evoked potentials. PLoS One. 10, 1–18. 10.1371/journal.pone.0140703 26479067 PMC4610694

[B48] Naseer N. Hong K. S. (2015). fNIRS-based brain–computer interfaces: a review. Front. Hum. Neurosci. 9, 3. 10.3389/fnhum.2015.00003 25674060 PMC4309034

[B49] Nguyen A. Alqurashi R. Raghebi Z. Banaei-kashani F. Halbower A. C. Dinh T.. (2016). “In-ear biosignal recording system: A wearable for automatic whole-night sleep staging,” in Proc. Workshop Wearable Syst. Appl, 19–24.

[B50] Nguyen T. Chung W. (2019). A single-channel ssvep-based bci speller using deep learning. IEEE Access. 7, 1752–1763. 10.1109/ACCESS.2018.2886759

[B51] Parini S. Maggi L. Turconi A. C. Andreoni G. (2009). A robust and self-paced BCI system based on a four class SSVEP paradigm: algorithms and protocols for a high-transfer-rate direct brain communication. Comput. Intell. Neurosci. 2009, 864564. 10.1155/2009/864564 19421416 PMC2676320

[B52] Podmore J. J. Breckon T. P. Aznan N. K. N. Connolly J. D. (2019). On the relative contribution of deep convolutional neural networks for SSVEPbased bio-signal decoding in BCI speller applications. IEEE Trans. Neural Syst. Rehabil. Eng. 27, 611–618. 10.1109/TNSRE.2019.2904791 30872236

[B53] Qin K. Wang R. Zhang Y. (2021). Filter bank-driven multivariate synchronization index for training-free SSVEP BCI. IEEE Trans. Neural Syst. Rehabil. Eng. 1−1. 10.1109/TNSRE.2021.3073165 33852389

[B54] Ravi A. Heydari Beni N. Manuel J. Jiang N. (2020). Comparing user-dependent and user-independent training of CNN for SSVEP BCI. J. Neural Eng. 17, 026028. 10.1088/1741-2552/ab6a67 31923910

[B55] Roy Y. Banville H. Albuquerque I. Gramfort A. Falk T. H. Faubert J. (2019). Deep learning-based electroencephalography analysis: a systematic review. J. Neural Eng. 16, 051001. 10.1088/1741-2552/ab260c 31151119

[B56] Suk H. I. Wee C. Y. Lee S. W. Shen D. (2016). State-space model with deep learning for functional dynamics estimation in resting-state fMRI. NeuroImage. 129, 292–307. 10.1016/j.neuroimage.2016.01.005 26774612 PMC5437848

[B57] Van Dun B. Wouters J. Moonen M. (2007). Improving auditory steadystate response detection using independent component analysis on multichannel EEG data. IEEE Trans. Biomed. Eng. 54, 1220–1230. 10.1109/TBME.2007.897327 17605353

[B58] Vialatte F. B. Maurice M. Dauwels J. Cichocki A. (2009). Steady-state visually evoked potentials: Focus on essential paradigms and future perspectives. Prog. Neurobiol. 90, 418–438. 10.1016/j.pneurobio.2009.11.005 19963032

[B59] Wang Y. Chen X. Gao X. Gao S. (2017). A benchmark dataset for SSVEP-based brain–computer interfaces. IEEE Trans. Neural Syst. Rehabil. Eng. 25, 1746–1752. 10.1109/TNSRE.2016.2627556 27849543

[B60] Wang Y. Gao X. Hong B. Jia C. Gao S. (2008). Brain-computer interfaces based on visual evoked potentials: feasibility of practical system design. IEEE EMB Mag. 27, 64–71. 10.1109/MEMB.2008.923958 18799392

[B61] Wang Y. Nakanishi M. Wang Y. T. Jung T. P. (2014). “Enhancing detection of steady-state visual evoked potentials using individual training data,” in Proc 36th Ann Int Conf IEEE Eng Med Biol Soc. 3037–3040. 25570631 10.1109/EMBC.2014.6944263

[B62] Wang Y. Wang R. Gao X. Hong B. Gao S. (2006). A practical VEP-based brain-computer interface. IEEE Trans. Neural Syst. Rehabil.Eng. 14, 234–239. 10.1109/TNSRE.2006.875576 16792302

[B63] Wang Y. Wang Y. T. Jung T. P. (2010). Visual stimulus design for high-rate SSVEP. Electron. Lett. 46, 1057–1058. 10.1049/el.2010.0923

[B64] Wang Y. T. Nakanishi M. Kappel S. L. Kidmose P. Mandic D. P. Wang Y.. (2015). “Developing an online steady-state visual evoked potential-based brain-computer interface system using EarEEG,” in Annu. Int. Conf. IEEE En.g Med. Biol. Soc. 2271–4. 26736745 10.1109/EMBC.2015.7318845

[B65] Wang Y. T. Nakanishi M. Wang Y. Wei C. S. Cheng C. K. Jung T. P. (2017). An online brain-computer interface based on SSVEPs measured from non-hair-bearing areas. IEEE Trans. Neural Syst. Rehabil. 25, 14–21. 10.1109/TNSRE.2016.2573819 27254871

[B66] Wang Y. T. Wang Y. Cheng C. K. Jung T. P. (2012). “Measuring steady state visual evoked potentials from non-hair-bearing areas,” in Proc.Annu. Int. Conf. IEEE Eng. Med. Biol. Soc. 1806–1809. 23366262 10.1109/EMBC.2012.6346301

[B67] Wang Y. T. Wang Y. Jung T. P. (2011). A cell-phone-based brain-computer interface for communication in daily life. J Neural Eng. 8, 025018. 10.1088/1741-2560/8/2/025018 21436517

[B68] Wolpaw J. R. Birbaumer N. Heetderks W. J. McFarland D. J. Peckham P. H. Schalk G.. (2000). Brain-computer interface technology: a review of the 1st international meeting. IEEE Trans. Rehabil. Eng. 8, 164–173. 10.1109/TRE.2000.847807 10896178

[B69] Wolpaw J. R. Birbaumer N. McFarland D. J. Pfurtscheller G. Vaughan T. M. (2002). Brain-computer interfaces for communication and control. Clin. Neurophysiol. 13, 767–791. 10.1016/S1388-2457(02)00057-3 12048038

[B70] Wolpaw J. R. McFarland D. J. Neat G. W. Forneris C. A. (1991). An EEGbased brain-computer interface for cursor control. Electroencephalogr. Clin. Neurophysiol. 78, 252–259. 10.1016/0013-4694(91)90040-B 1707798

[B71] Xu M. Chen L. Zhang L. Qi H. Ma L. Tang J.. (2014). A visual parallel-BCI speller based on the time-frequency coding strategy. J. Neural Eng. 11, 026014. 10.1088/1741-2560/11/2/026014 24608672

[B72] Yin E. Zhou Z. Jiang J. Chen F. Liu Y. Hu D. (2013). A novel hybrid BCI speller based on the incorporation of SSVEP into the P300 paradigm. J. Neural Eng. 10, 026012. 10.1088/1741-2560/10/2/026012 23429035

[B73] Zhang X. Yao L. Wang X. Monaghan J. McAlpine D. Zhang Y. (2021). A survey on deep learning-based non-invasive brain signals: recent advances and new frontiers. J. Neural Eng. 18, 031002. 10.1088/1741-2552/abc902 33171452

[B74] Zhang Y. Zhou G. Jin J. Wang M. Wang X. Cichocki A. (2013). L1-Regularized multiway canonical correlation analysis for SSVEP-based BCI. IEEE Trans. Neural Syst. Rehabil. Eng. 21, 887–896. 10.1109/TNSRE.2013.2279680 24122565

[B75] Zhang Y. Zhou G. Jin J. Wang X. Cichocki A. (2014). Frequency recognition in SSVEP-based BCI using multiset canonical correlation analysis. Int. J. Neural Syst. 24, 1450013. 10.1142/S0129065714500130 24694168

[B76] Zhang Y. Zhou G. Zhao Q. Onishi A. Jin J. Wang X.. (2011). “Multiway canonical correlation analysis for frequency components recognition in SSVEP-based BCIs,” in Proc 18th Int Conf Neural Inform Process. 287–295.

[B77] Zhu Y. Li Y. Lu J. Li P. (2021). EEGNet With Ensemble Learning to Improve the Cross-Session Classification of SSVEP Based BCI From Ear-EEG. IEEE Access. 9, 15295–15303. 10.1109/ACCESS.2021.3052656

